# Impact of capillary invasion on the prognosis of gastric adenocarcinoma patients: A retrospective cohort study

**DOI:** 10.18632/oncotarget.9101

**Published:** 2016-04-29

**Authors:** Lian Xue, Xiao-Long Chen, Pan-Pan Lin, Yuan-Wei Xu, Wei-Han Zhang, Kai Liu, Xin-Zu Chen, Kun Yang, Bo Zhang, Zhi-Xin Chen, Jia-Ping Chen, Zong-Guang Zhou, Jian-Kun Hu

**Affiliations:** ^1^ Department of Gastrointestinal Surgery, West China Hospital, Sichuan University, China; ^2^ Laboratory of Gastric Cancer, State Key Laboratory of Biotherapy/Collaborative Innovation Center of Biotherapy and Cancer Center, West China Hospital, Sichuan University, China; ^3^ Laboratory of Digestive Surgery, State Key Laboratory of Biotherapy/Collaborative Innovation Center of Biotherapy and Cancer Center West China Hospital, Sichuan University, China; ^4^ West China School of Medicine, Sichuan University, China

**Keywords:** gastric adenocarcinoma, capillary invasion, prognosis, nomogram

## Abstract

Capillary invasion (CI) has been found to play an important role in metastasis and recurrence of gastric adenocarcinoma (GAC). However, the prognostic significance of CI is still controversial. From January 2005 to December 2011, 1398 patients with GAC who underwent gastrectomy were retrospectively enrolled and divided into CI (+) and CI (−) groups. Clinicopathological features and survival outcomes were compared between these groups. In our study, 227 (16.2%) patients were CI (+). Patients with CI (+) had significantly more advanced tumors and worse prognosis than those with CI (−) (*p* < 0.001). CI was demonstrated as an independent prognostic factor (*p* = 0.023) in patients with GAC. When stratified by TNM stage, the prognosis of CI (+) group in stage III was remarkably worse than CI (−) group (*p* = 0.006), while the differences were not significant in stage I–II and stage IV (both *p* > 0.05). The nomograms indicated that CI was part of the individual prognostic prediction system. The predictive accuracy of CI and other characteristics was better than TNM alone (*p* < 0.001). Our finding suggested that CI was an independent prognostic factor in patients with GAC, and the nomogram based on CI and other clinicopathological factors was a valuable and accurate tool in individual prognostic prediction.

## INTRODUCTION

Gastric adenocarcinoma (GAC) is one of the most common malignant cancers in the world, especially in East Asian countries like China, Korea and Japan [1]. Although prognosis of patients with GAC has been improved by early detection, chemoradiotherapy and radical lymphadenectomy, the mortality of GAC is still high, which mainly caused by recurrence and metastasis [2, 3]. Additionally, even among patients with the same TNM stage, the long-term prognosis might be different. Therefore, it's necessary to explore new factors besides TNM stage to accurately predict prognosis in patients with GAC.

Capillary invasion (CI) is defined as lymphatic invasion and/or venous invasion according to Japanese classification of gastric carcinoma [4]. With tumor progression, tumor cells can invade blood vessels and lymphatic vessels nearby. Some studies had found that lymphatic vessels and blood vessels may help tumor metastasis and recurrence [5, 6]. CI had been regarded as an adverse prognostic factor in some tumors like esophageal squamous cell carcinoma, lung tumor and breast cancer [7–9]. Although there were several studies on the CI in gastric cancer, the prognostic significance of CI hadn't been fully verified and needs further demonstration [10–13]. The aim of this study was to find out the prognostic significance of CI in patients with GAC.

## RESULTS

### The relationship between CI and clinicopathological features

There were 1398 patients in our study with 227 (16.2%) patients in CI (+) group and 1171 (83.8%) patients in CI (−) group. Of all the 1398 patients, 422 (30.2%) were women while 976 (69.8%) were men. The average age was 57.1years, ranging from 19 to 86 years.

Comparison of clinicopathological characteristics between CI (+) and CI (−) groups were shown in Table 1. Patients with CI (+) had significantly more tumors with UML location (*p* = 0.020), poorly/undifferentiated differentiation grade (*p* < 0.001), larger tumor size (*p* < 0.001), more advanced macroscopic type (*p* < 0.001) and TNM stage (*p* < 0.001) than those with CI (−). On the other hand, the relationship between CI (+) and age (*p* = 0.451), gender (*p* = 0.934) was not found. To identify the independent risk factors for CI, multivariate analyses were performed in our study (Table 2). By logistic regression analysis, we found that CI was significantly correlated to differentiation grade (*p* = 0.009) and pN stage (*p* < 0.001).

**Table 1:** Comparison of clinicopathological features between capillary invasion (CI) positive and negative group

	Categories	CI (+)	CI (−)	*P* value
		*n* = 227 (%)	*n* = 1171(%)	
Age	< 60 years	121 (53.3)	656 (56.0)	0.451
	≥ 60 years	106 (46.7)	515 (44.0)	
Gender	Male	159 (70.0)	817 (69.8)	0.934
	Female	68 (30.0)	354 (30.2)	
Longitudinal location	U	53 (23.3)	285 (24.3)	0.020
	M	53 (23.3)	200 (17.1)	
	L	114 (50.2)	671 (57.3)	
	UML	7 (3.1)	15 (1.3)	
Differentiation grade	Well/Moderately	21 (9.3)	270 (23.1)	< 0.001
	Poorly/undifferentiated	206 (90.7)	901 (76.9)	
Macroscopic type	Type 0	11 (4.8)	202 (17.3)	< 0.001
	Type 1	9 (4.0)	47 (4.0)	
	Type 2	115 (50.7)	546 (46.6)	
	Type 3	75 (33.0)	321 (27.4)	
	Type 4	17 (7.5)	55 (4.7)	
Tumor size (cm)	Mean ± SD	5.6 ± 3.2	4.7 ± 2.6	< 0.001
	< 5 cm	92 (40.5)	630 (53.8)	
	≥ 5 cm	135 (59.5)	541 (46.2)	
T stage	T1	17 (7.5)	312 (26.6)	< 0.001
	T2	34 (15.0)	165 (14.1)	
	T3	17 (7.5)	101 (8.6)	
	T4	159 (70.0)	593 (50.6)	
N stage	N0	34 (15.0)	461 (39.4)	< 0.001
	N1	29 (12.8)	204 (17.4)	
	N2	33 (14.5)	191 (16.3)	
	N3	131 (57.7)	315 (26.9)	
M stage	M0	202 (89.0)	1115 (95.2)	< 0.001
	M1	25 (11.0)	56 (4.8)	
TNM stage	I	26 (11.5)	376 (32.1)	< 0.001
	II	32 (14.1)	228 (19.5)	
	III	144 (63.4)	511 (43.6)	
	IV	25 (11.0)	56 (4.8)	
Adjuvant therapy	Yes	104 (45.8)	504 (43.0)	0.440
	No	123 (54.2)	667 (57.0)	

Abbreviations: SD: standard deviation, U: upper, M: middle, L: lower.

**Table 2:** Multivariate analysis of risk factors for capillary invasion (CI)

Factors	Adjusted OR	95% CI	*P* value
Age			0.196
Gender			0.786
Longitudinal location			0.954
Differentiation grade	1.915	1.176–3.116	0.009
Macroscopic type			0.352
Tumor size (cm)			0.969
T stage			0.299
N stage	1.690	1.483–1.925	< 0.001
M stage			0.333

Abbreviations: SD: standard deviation.

Because of the different constituent ratio of TNM stage in CI (+) and CI (−) groups which made some biases to compare the prognosis between these two groups directly, we then conducted the subgroup analyses according to different TNM stages: stage I–II (*n* = 662, 47.4%), stage III (*n* = 655, 46.9%) and stage IV (*n* = 81, 5.7%). The rates of CI (+) were 8.8%, 22.0% and 30.9% in TNM I–II, III and IV subpopulation respectively. In the subgroup of TNM stage I–II, we found that there were more tumors with poorly differentiation grade (*p* = 0.026), more advanced pT stage (*p* < 0.001) and pN stage (*p* = 0.013) in CI (+) group than those in CI (−) group (Table 3). With regard to TNM stage III subgroup, the results demonstrated that there were more patients with N2 and N3 stage (*p* < 0.001) tumors in CI (+) group than those in CI (−) group. However, in TNM IV subgroup, there were no significant differences between patients with CI (+) and CI (−).

**Table 3:** Clinicopathological features of capillary invasion (CI) negative and positive groups stratified by TNM stage

		Stage I–II (*n* = 662, 47.4%)						Stage IV (*n* = 81, 5.7%)		
		Negative (%)	Positive (%)	*P* value	Negative (%)	Positive (%)	*P* value	Negative (%)	Positive (%)	*P* value
		(*n* = 604)	(*n* = 58)		(*n* = 511)	(*n* = 144)		(*n* = 56)	(*n* = 25)	
Age (year)	Mean ± SD	56.8 ± 11.9	57.2 ± 12.9	0.807	57.0 ± 11.3	58.4 ± 11.7	0.201	57.0 ± 12.5	56.8 ± 11.1	0.936
	< 60	338 (56)	31 (53.4)	0.713	289 (56.6)	74 (51.4)	0.271	29 (51.8)	16 (64.0)	0.307
	≥ 60	266 (44)	27 (46.6)		222 (43.4)	70 (48.6)		27 (48.2)	9 (36.0)	
Gender	Male	424 (70.2)	34 (58.6)	0.068	357 (69.9)	110 (76.4)	0.126	36 (64.3)	15 (60.0)	0.712
	Female	180 (29.8)	24 (41.4)		154 (30.1)	34 (23.6)		20 (35.7)	10 (40.0)	
Longitudinal	U	110 (18.2)	15 (25.9)	0.201	158 (30.9)	34 (23.6)	0.128	17 (30.4)	4 (16.0)	0.115
location	M	96 (15.9)	7 (12.1)		95 (18.6)	36 (25)		9 (16.1)	10 (40.0)	
	L	396 (65.6)	35 (60.3)		251 (49.1)	70 (48.6)		24 (42.9)	9 (36.0)	
	UML	2 (0.3)	1 (1.7)		7 (1.4)	4 (2.8)		6 (10.7)	2 (8.0)	
Macroscopic	Type 0	196 (32.5)	8 (13.8)	0.067	6 (1.2)	2 (1.4)	0.988	0 (0)	1 (4.0)	0.601
type	Type 1	32 (5.3)	4 (6.9)		11 (2.2)	4 (2.8)		4 (7.1)	1 (4.0)	
	Type 2	266 (44)	32 (55.2)		255 (49.9)	70 (48.6)		25 (44.6)	13 (52.0)	
	Type 3	104 (17.2)	13 (22.4)		196 (38.4)	55 (38.2)		21 (37.5)	7 (28.0)	
	Type 4	6 (1)	1 (1.7)		43 (8.4)	13 (9.0)		6 (10.7)	3 (12.0)	
Differentiation	Well/Moderately	201 (33.3)	11 (19.0)	0.026	62 (12.1)	10 (6.9)	0.079	7 (12.5)	0 (0)	0.093
grade	Poorly	403 (66.7)	47 (81.0)		449 (87.9)	134 (93.1)		49 (87.5)	25 (100.0)	
Tumor size	Mean ± SD	3.5 ± 2.0	3.9 ± 2.3	0.150	5.8 ± 2.5	6.0 ± 2.9	0.309	7.1 ± 3.4	7.2 ± 4.5	0.870
(cm)	< 5	456 (75.5)	43 (74.1)	0.819	162 (31.7)	43 (29.9)	0.674	12 (21.4)	6 (24.0)	0.797
	≥ 5	148 (24.5)	15 (25.9)		349 (68.3)	101 (70.1)		44 (78.6)	19 (76.0)	
pT stage	T1	312 (51.7)	16 (27.6)	< 0.001	0 (0)	0 (0)	0.861	0 (0)	1 (4.0)	0.178
	T2	138 (22.8)	27 (46.6)		27 (5.3)	6 (4.2)		0 (0)	1 (4.0)	
	T3	58 (9.6)	5 (8.6)		41 (8.0)	12 (8.3)		2 (3.6)	0 (0)	
	T4	96 (15.9)	10 (17.2)		443 (86.7)	126 (87.5)		54 (96.4)	23 (92.0)	
pN stage	N0	455 (75.3)	33 (56.9)	0.013	4 (0.8)	1 (0.7)	< 0.001	2 (3.6)	0 (0)	0.547
	N1	112 (18.5)	19 (32.8)		90 (17.6)	10 (6.9)		2 (3.6)	0 (0)	
	N2	31 (5.1)	4 (6.9)		155 (30.3)	25 (17.4)		5 (8.9)	4 (16.0)	
	N3	6 (1.0)	2 (3.4)		262 (51.3)	108 (75.0)		47 (83.9)	21 (84.0)	
M stage	M0	604 (100.0)	58 (100.0)	—	511 (100.0)	144 (100.0)	—	0 (0)	0 (0)	—
	M1	0 (0)	0 (0)		0 (0)	0 (0)		56 (100.0)	25 (100.0)	
TNM stage	I	376 (62.3)	26 (44.8)	0.009	0 (0)	0 (0)	—	0 (0)	0 (0)	—
	II	228 (37.7)	32 (55.2)		0 (0)	0 (0)		0 (0)	0 (0)	
	III	—	—		511 (100.0)	144 (100.0)		0 (0)	0 (0)	
	IV	—	—		0 (0)	0 (0)		56 (100.0)	25 (100.0)	

### Prognostic significance of CI

**Figure 1:**
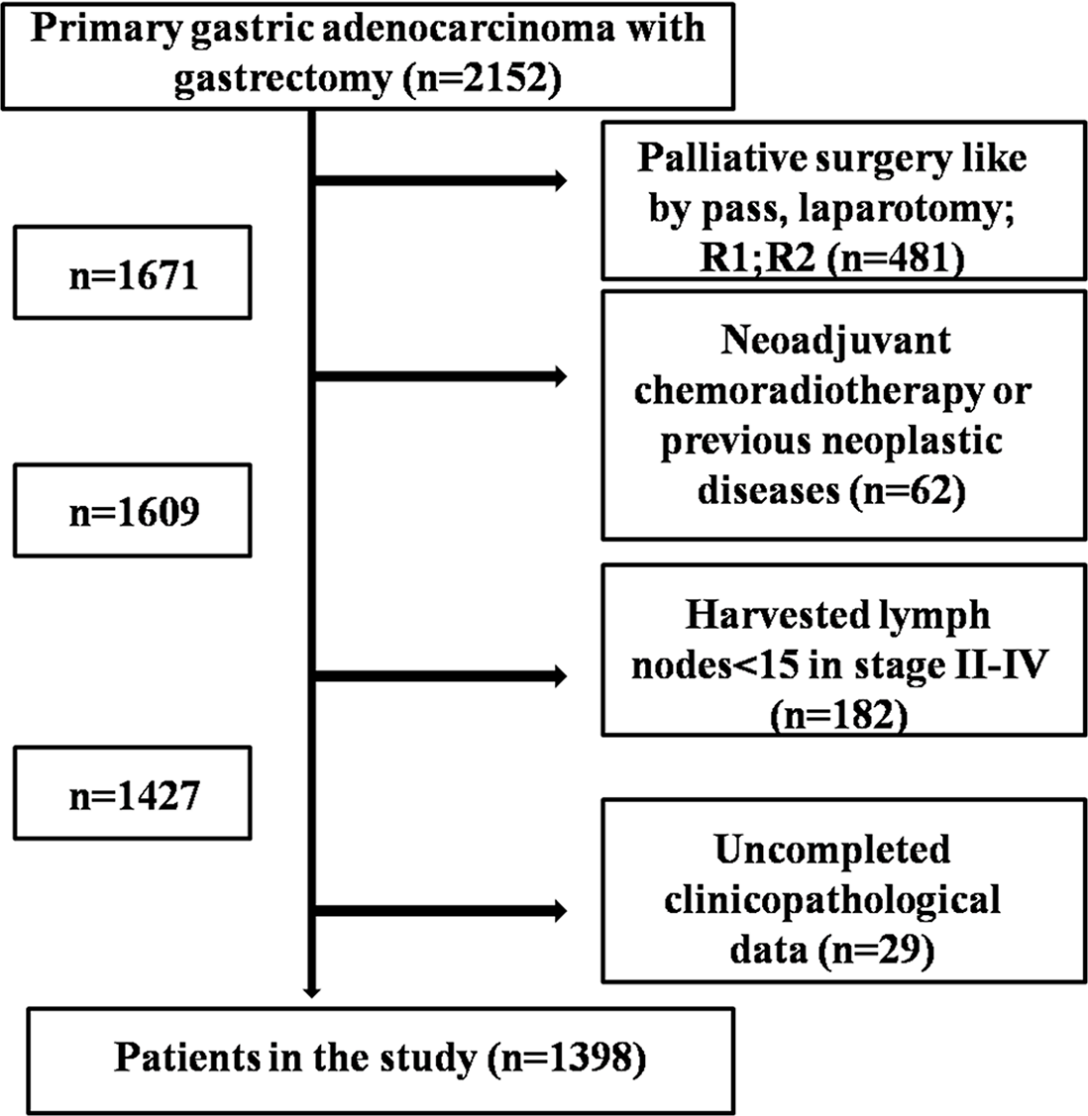
Flow chart of the patients

Finally, 1277 (91.3%) patients with follow-up data were included in the survival analysis. The median survival time of patients with CI (+) or CI (−) was 46.4 and 96.0 months respectively. Three-year survival rates were 55.4% and 74.4% in CI (+) and CI (−) respectively. In univariate analysis by Kaplan-Meier curve (Table 4), age (*p* < 0.001), longitudinal location (*p* < 0.001), differentiation grade (*p* < 0.001), macroscopic type (*p* < 0.001), tumor size (*p* < 0.001), capillary invasion (*p* < 0.001), T stage (*p* < 0.001), N stage (*p* < 0.001), M stage (*p* < 0.001) and TNM stage (*p* < 0.001) were closely associated with overall survival of gastric adenocarcinoma patients. Patients with CI (+) had significant worse prognosis than those with CI (−) in Kaplan-Meier analysis (*p* < 0.001, Figure 1). Additionally, we performed multivariate analysis with Cox regression to further evaluate the prognostic significance of CI and other clinicopathological factors (Table 4), and we found that CI (*p* = 0.023, HR = 1.270, 95% confidence interval [1.034−1.560]) was an independent prognostic factor, as well as other clinicopathological factors like age (*p* = 0.002), tumor size (*p* < 0.001) and TNM stage (*p* < 0.001).

**Table 4:** Univariate and multivariate Cox analysis for prognostic factors

Risk factors	Categories	Univariate analysis *P* value	Multivariate analysis		
			HR	95% CI	*P* value
Age (years)	< 60	< 0.001	1.306	1.104–1.546	0.002
	≥ 60				
Gender	Male	0.074			0.345
	Female				
Longitudinal location	U	< 0.001			0.139
	M				
	L				
	UML				
Differentiation grade	Well/Moderate differentiated	< 0.001			0.692
	Poor/undifferentiated				
Macroscopic type	Type 0	< 0.001			0.198
	Type 1				
	Type 2				
	Type 3				
	Type 4				
Tumor size	< 5 cm	< 0.001	1.425	1.183–1.717	< 0.001
	≥ 5 cm				
Capillary invasion	Positive	< 0.001	1.270	1.034–1.560	0.023
	Negative				
T stage	T1	< 0.001	—	—	—
	T2				
	T3				
	T4				
N stage	N0	< 0.001	—	—	—
	N1				
	N2				
	N3				
M stage	M0	< 0.001	—	—	—
	M1				
TNM stage	I	< 0.001	1.953	1.745–2.185	< 0.001
	II				
	III				
	IV				

**Figure 2:**
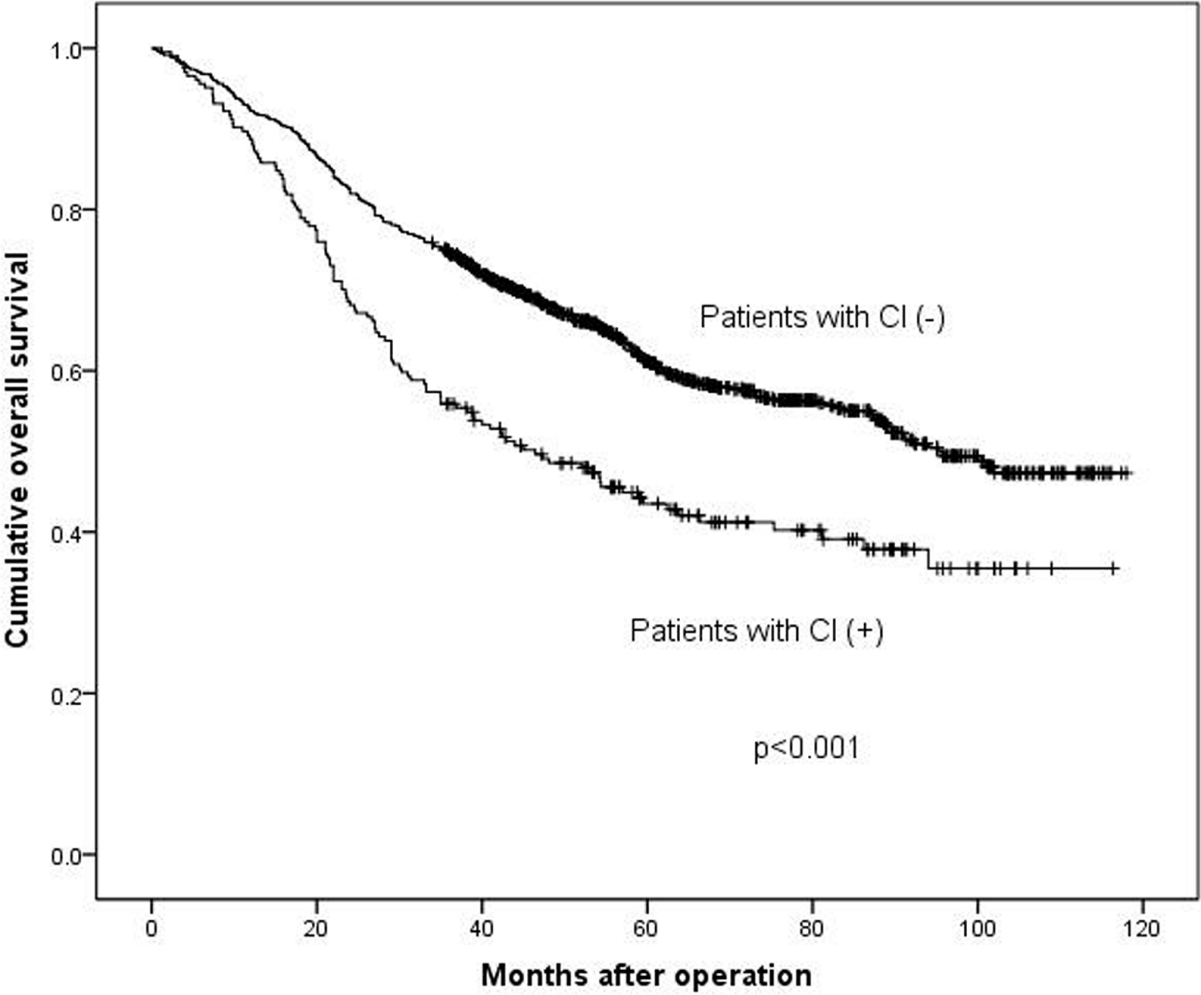
Survival analysis between patients with CI (+) and CI (−)

**Figure 3:**
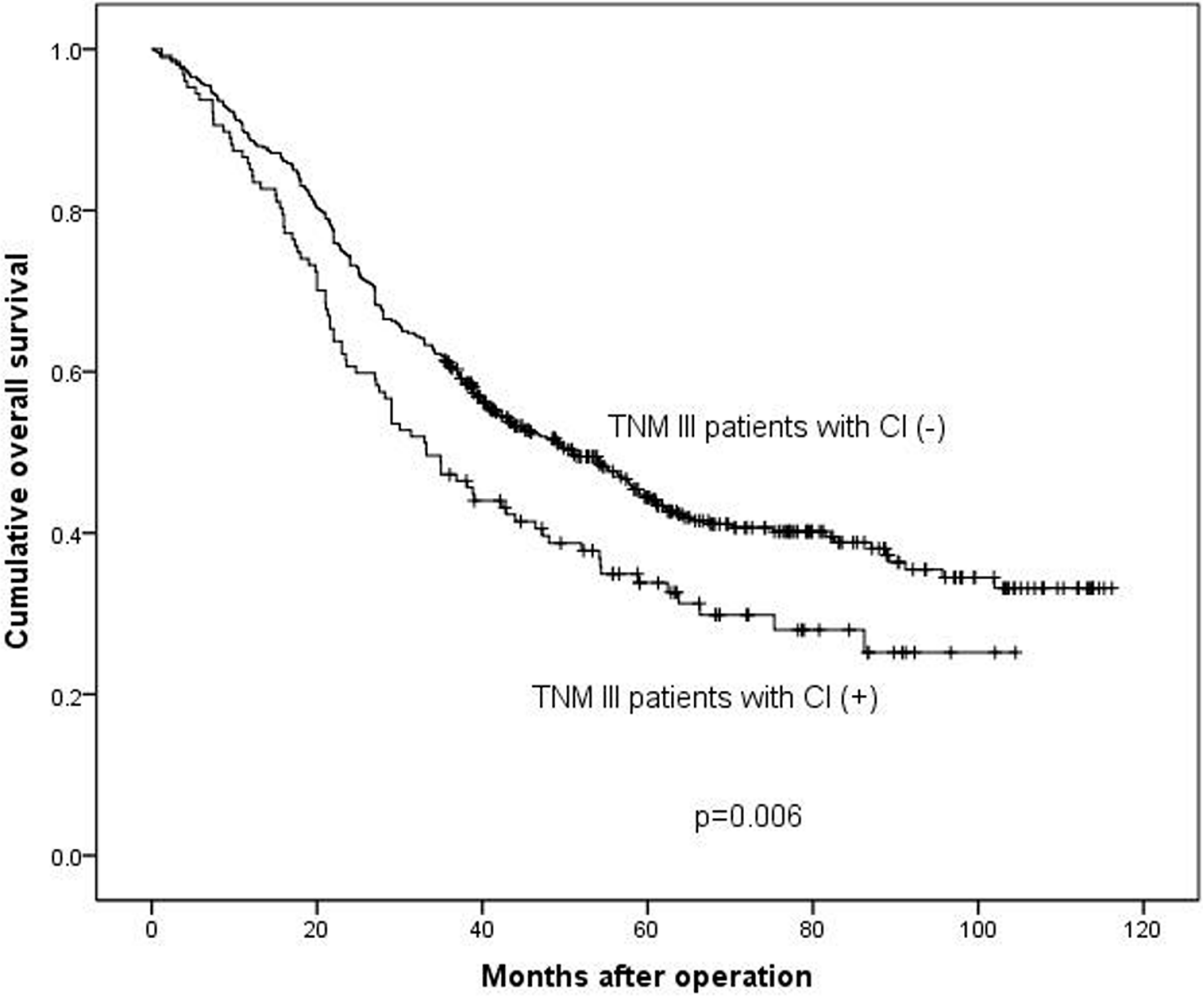
Survival analysis between TNM III stage patients with CI (+) and CI (−)

**Figure 4:**
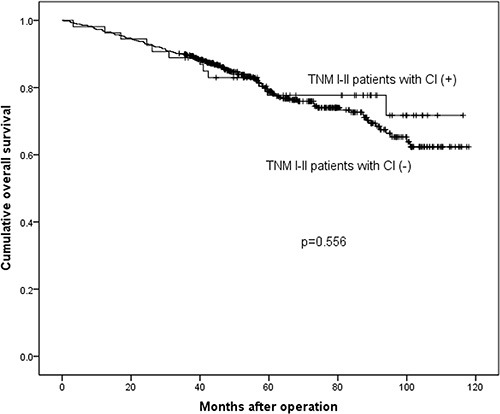
Survival analysis between TNM I–II stage patients with CI (+) and CI (−)

Survival analyses were calculated among these different subgroups respectively. The median survival time was 117.0 months and 116.0 months in stage I–II, 51.3 months and 33.8 months in stage III, 20.9 months and 24.5 months in stage IV in CI (−) and CI (+) groups respectively. The 3-year survival rates were 89.6% and 88.9% in stage I–II, 60.3% and 46.5% in stage III, 34.7% and 26.1% in stage IV in CI (−) and CI (+) groups respectively. The results demonstrated that the prognosis of CI (+) group in stage III (*p* = 0.006, Figure 2) was significantly worse than that of CI (−) group, while in stage I–II (*p* = 0.556, Figure 3) and stage IV (*p* = 0.904, Figure 4), the difference wasn't remarkable.

### Nomogram based on CI

**Figure 5:**
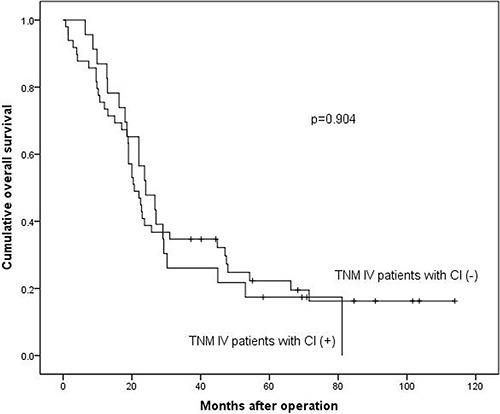
Survival analysis between TNM IV stage patients with CI (+) and CI (−)

**Figure 6:**
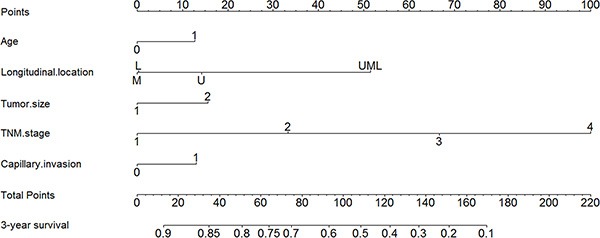
Nomogram for gastric adenocarcinoma patients

We also used nomogram to predict 3-year overall survival rate of individual patients. Age, tumor size, TNM stage and CI (*p* = 0.015, hazard ratio 1.292, 95% confidence interval: 1.052, 1.587) were selected in the nomogram (Figure 5). The nomograms indicated that CI was part of the individual prognostic prediction system. The results of the nomograms were similar to those of aforementioned multivariate analyses. The calibration curves of nomograms showed that the predictive probability of 3-year survival was very closely to the actual 3-year survival (Figure 6).

Subsequently, we compared the predictive accuracy of prognosis between the nomogram and TNM staging system in the study. The C-indexes of nomograms were 0.718 (95% CI 0.696 0.740), compared with 0.689 (95% CI 0.669, 0.709) of TNM staging system. The differences between nomograms and TNM staging system were significant (*p* < 0.001).

## DISCUSSION

CI included lymphatic invasion and/or venous invasion according to Japanese classification of gastric carcinoma [4]. Many studies had discussed the prognostic significance of lymphatic vessel invasion and blood vessel invasion respectively [9–11], however, the studies which combined these two factors together to explore the prognostic significance in GAC were few [12]. In this retrospective study, we tried to find out the prognostic significance of CI in GAC.

The presence of CI in gastric cancer was reported from 5.4% to 86% [10–16]. This could be due to the different populations included in different studies and different methods such as retrospective studies or experimental studies by HE staining and immunohistochemical staining. We observed the presence of CI in gastric adenocarcinoma was 16.2% in our patients. The incidence of CI was found to increase with the progression of tumor. There was a significant positive relationship between CI and TNM stage which was similar to previous study [10]. In our study, we found that gastric adenocarcinoma patients with CI (+) had more poorly differentiated tumors, larger tumor size and more advanced macroscopic type and TNM stage than CI (−) group, which was similar to previous researches [17, 18]. We concluded that CI was associated with tumor aggressiveness. And we also found that CI was significantly associated with differentiation grade and pN stage in multivariate analysis. One explanation to this was that when the tumor cells metastasis to the lymph nodes, it would pass through the lymphatic vessels, and the probability of lymphatic vessels invasion would increase significantly. Our results were similar to previous study and could also suggest the viewpoint that presence of CI can be an indication for a more extensive surgical resection [10]. Del Casar reported that lymphatic and blood vessels invasion was closely associated with undifferentiated histological subtype [10]. And in our study, we also found the similar results. The reason might be that tumor cells in poorly differentiated tumors tended to metastasis more easily than well differentiated tumors, which could also increase the probability of blood vessels and lymphatic vessels invasion.

Some studies reported that CI was an independent prognostic factor by multivariate survival analysis [10, 11], while other study demonstrated that CI didn't show prognostic significance as an independent prognostic factor for all patients with gastric carcinoma [19]. We found that the presence of CI was an independent prognostic factor in patients with GAC (*p* = 0.023). We also found that the patients with CI (+) had worse prognosis than CI (−) patients (*p* < 0.001), which was consistent with previous studies [17, 20]. The reason might be that patients with CI (+) had more advanced TNM stage tumors. In order to eliminate the bias caused by different constituent ratio of TNM stage in CI (+) and CI (−) groups, we divided patients into three subgroups (TNM I–II subgroup, III subgroup and IV subgroup) according to their TNM stage to minimize the influence of survival outcomes by TNM stage. After the stratification, we further compared the prognosis between patients with CI (+) and CI (−) in each subgroups. The prognosis didn't show significant difference between TNM I–II subgroup (*p* = 0.556) and TNM IV subgroup (*p* = 0.904). However, in TNM III stage subgroup, we found that patients with CI had significantly worse prognosis than the ones without CI (*p* = 0.006). The possible explanation might be that the prognosis of patients with TNM I–II stage is much better than patients with III or IV TNM stages, and maybe the adverse effect of CI can't be completely reflected in TNM I–II stage and the CI may not influence the prognosis of patients with TNM I–II severely. Thus the prognoses of CI (+) and CI (−) patients in TNM I-II subgroup were not significantly different. Similarly, the prognosis of patients with TNM IV stage is too worse to be influenced by CI. Thus, CI could show its adverse prognostic value only in gastric adenocarcinoma patients with TNM III stage. Although distant metastasis didn't appear in patients with TNM III stage, we still should pay attention to TNM III stage patients with CI (+) for their worse prognosis than patients with CI (−).

Nomogram is a visualized method to predict the prognosis of individual patient on the basis of some valuable parameters. According to nomogram, the prognosis of individual patients can be predicted well. In our study, we also compared the predictive accuracy between nomogram and TNM staging system, and the results showed that nomogram with CI and other characteristics was better than TNM alone. However, we still considered TNM stage as one of the most important prognostic factors of GAC, but more importantly, other indexes like CI should be also noticed.

In this retrospective study, patients with GAC were enrolled to discuss the prognostic significance of CI. Patients with CI (+) had more advanced TNM stage tumors and worse prognosis than those with CI (−), especially in TNM III stage. And the CI turned out to be an independent prognostic factor for patients with GAC. Although TNM stage is the most important factor to predict survival of patients with GAC, other indexes like CI shouldn't be ignored neither. However, the present findings are retrospective and it's necessary to carry out prospective, randomized, controlled study to examine the prognostic value of CI in GAC.

## MATERIALS AND METHODS

The West China Hospital research ethics committee approved retrospective analysis of anonymous data. Signed patient informed consent was waived per the committee approval, because it was a retrospective analysis.

### Patients

**Figure 7:**
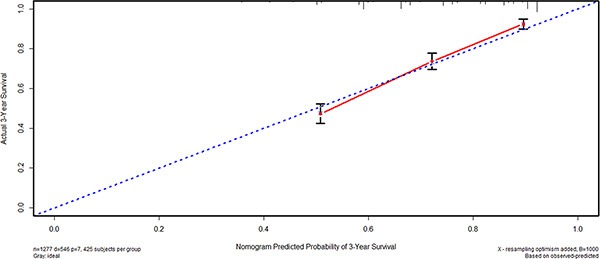
Calibration curve for gastric adenocarcinoma patients

In our study, we enrolled patients with GAC who underwent gastrectomy with curative intent from Department of Gastrointestinal Surgery, West China Hospital from January 2005 to December 2011. Patients with neoadjuvant chemoradiotherapy, previous neoplastic diseases and uncompleted clinicopathological data were excluded. To eliminate the impact of insufficient lymphadenectomy, we also excluded TNM stage II–IV patients with less than 15 lymph nodes harvested in surgery. Finally, there were 1398 patients in our study (Figure 7). The clinicopathological features of these patients such as tumor size, differentiation grade, macroscopic type and TNM stage which was defined according to Japanese classification of gastric carcinoma by JGCA were recorded [4]. Patients were divided according to whether they had capillary invasion: CI (+) and CI (−) groups.

### Capillary invasion

CI including lymphatic invasion and venous invasion was defined as the presence of tumor cells in the lumen of lymphatic/venous vessels, which were lined by endothelial cells. The histopathology reports of the specimens were made by experienced pathologists from West China Hospital.

### Follow-up

Follow-up information was updated to January 2015. Regular outpatient visit was chosen as the main method for follow-up, while telephones and mails were selected as two main supplementary methods. The main reasons for the loss of follow-up were the changes of phone number or home address and refusal of re-examination in our hospital.

### Statistical analysis

All the statistical analyses were performed with the statistical software SPSS 19.0 (SPSS®, Chicago, Illinois, USA). All continuous variables were presented as mean ± standard deviation (SD). Unordered categorical variable and ranked data was analyzed by chi-square test and rank sum test (Mann-Whitney *U* test), respectively. Student's *t*-testwas used to analyze continuous data if homogeneity of variance and normal distribution. If not, rank sum test was used. Logistic regression was used in multivariate correlation analysis. Kaplan-Meier method and life-table method were used to calculate the cumulative survival rate. Log-rank test and Cox's proportional hazard regression model were conducted for univariate and multivariate survival analyses, respectively. Nomogram and calibration curve were performed through R for Windows (Version 3.2.0, R Foundation for Statistical Computing) with the package of Regression Modeling Strategies (rms), in which the variables were selected according to the model by Akaike information criterion in a stepwise algorithm. Comparisons between the nomogram and TNM staging systems were performed with the package of Harrell Miscellaneous (Hmisc) and were evaluated by the C-index, with the meaning of that the larger the C-index, the more accurate was the prognostic prediction. *P* value less than 0.05 was considered as statistical significance.
